# Quality Improvement Project to Reduce the Anticholinergic Burden in a Rehabilitation Unit

**DOI:** 10.1192/bjo.2024.387

**Published:** 2024-08-01

**Authors:** Suryanarayana Kakkilaya, Shirley Morton, Sue Evans

**Affiliations:** 1 Draper House, St Helens, United Kingdom; 2 Krinvest Care, St Helens, United Kingdom

## Abstract

**Aims:**

We conducted a quality improvement project in a female rehabilitation unit, with an aim to reduce the total anticholinergic burden.

**Methods:**

Most patients were reluctant to change the medications that they have been taking for long time. Hospital staff were also concerned about the potential risk of destabilising the mental health of patients who are currently stable.

As a first step, we used ACB (Anticholinergic Burden) calculator to calculate ACB score

We agreed on a realistic safe target. We decided not to include patients who are on clozapine. Information related to anticholinergic burden was shared with nursing team and staff members. This was discussed in MDT meetings to answer any questions.

Team collaboratively created an information leaflet, including an easy read version. Group sessions and 1:1 sessions were arranged with patients to discuss the potential side effects.

Medication changes were carried out following a consultation with patients.

**Results:**

ACB score of all 15 patients were over 3. One patient is over the age of 65. Five patients scored more than 10 on total ACB score. Two patients were on clozapine.

Promethazine, procyclidine, hyoscine hydrobromide, oxybutynin and clozapine were causing most of the anticholinergic burden.

We decided not to change medications of two patients who were on clozapine. For the remaining patients procyclidine and promethazine were reviewed and stopped following a consultation. All 12 patients' ACB score is now less than 10. There has been a reduction of 3–6 points.

**Conclusion:**

This project has helped in reducing the ACB burden successfully. Promethazine with an ACB score of 3 was stopped for all patients. Some patients received promazine instead of promethazine. Procyclidine has been stopped for several patients and for some patients it has been changed from regular to PRN (to take when required). Consideration has been given to reduce the dose of typical antipsychotic medication instead of using procyclidine to treat extrapyramidal side effects.

Providing information and then reviewing the prescription of promethazine and procyclidine has resulted in significant reduction in the total ACB score.

